# Dataset of red light induced pupil constriction superimposed on post-illumination pupil response

**DOI:** 10.1016/j.dib.2016.08.003

**Published:** 2016-08-04

**Authors:** Shaobo Lei, Herbert C. Goltz, Jaime C. Sklar, Agnes M.F. Wong

**Affiliations:** a Program in Neurosciences and Mental Health, University of Toronto, Toronto, Canada; b Department of Ophthalmology and Vision Sciences, University of Toronto, Toronto, Canada; c The Hospital for Sick Children, University of Toronto, Toronto, Canada

**Keywords:** Pupil light reflex, Chromatic pupillometry, Melanopsin, Post-illumination pupil response

## Abstract

We collected and analyzed pupil diameter data from of 7 visually normal participants to compare the maximum pupil constriction (MPC) induced by “Red Only” vs. “Blue+Red” visual stimulation conditions.

The “Red Only” condition consisted of red light (640±10 nm) stimuli of variable intensity and duration presented to dark-adapted eyes with pupils at resting state. This condition stimulates the cone-driven activity of the intrinsically photosensitive retinal ganglion cells (ipRGC). The “Blue+Red” condition consisted of the same red light stimulus presented during ongoing blue (470±17 nm) light-induced post-illumination pupil response (PIPR), representing the cone-driven ipRGC activity superimposed on the melanopsin-driven intrinsic activity of the ipRGCs (“The Absence of Attenuating Effect of Red light Exposure on Pre-existing Melanopsin-Driven Post-illumination Pupil Response” Lei et al. (2016) [1]).

MPC induced by the “Red Only” condition was compared with the MPC induced by the “Blue+Red” condition by multiple paired sample *t*-tests with Bonferroni correction.

**Specifications Table**

**Table:** 

Subject area	*Biology*
More specific subject area	*Ophthalmology and Vision Science*
Type of data	*Graphs*
How data were acquired	*Real time pupillometry recording*
Data format	*Analyzed*
Experimental factors	*Visual stimulation of variable wavelength, luminance and duration*
Experimental features	*Pupillary light response to visual stimulation*
Data source location	*Toronto, Ontario, Canada*
Data accessibility	*Data are included in this article*

**Value of the data**

• The presented data demonstrate the temporal integration of the extrinsic cone-driven activity of ipRGCs and the melanopsin-driven intrinsic activity of ipRGCs in an *in vivo* fashion.
• The presented data can serve as a benchmark for other researchers who are interested in investigating the interaction between extrinsic and intrinsic ipRGC activity.
• The presented data may be useful in the development of chromatic pupillometry as an *in vivo* clinical assessment of ipRGC function.

## 1 Data

**Fig. 1:**
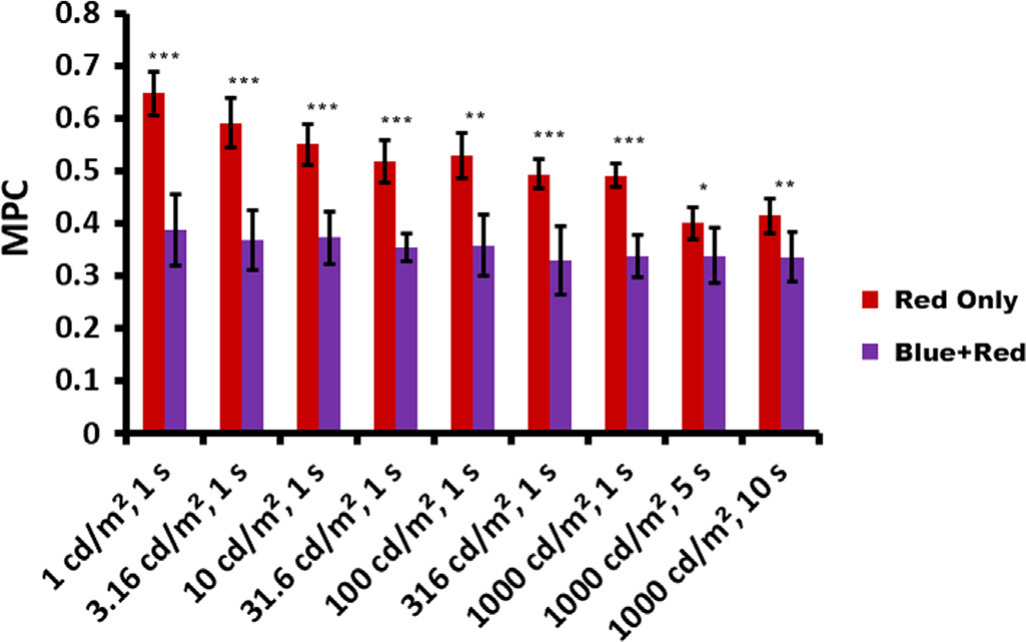
Comparisons of maximum pupil constriction (MPC) induced by “Red Only” stimuli vs. red stimuli presented during ongoing blue-light-induced PIPR (Blue+Red). Smaller values represent greater pupil constriction. Error bars represent ±1 standard deviation. Compared to the “red only” reference condition, red light exposure presented after the melanopsin-activating blue light stimulation induced greater MPC; all pair-wise comparisons reached statistical significance (*df*=6, *p<0.05, **p<0.01, ***p<0.001, paired sample *t*-test Bonferroni corrected for multiple comparisons). (For interpretation of the references to color in this figure legend, the reader is referred to the web version of this article).

Maximum pupil constrictions (MPCs) in response to 9 intensity/duration steps of “Red Only” light stimulation (1, 3.16, 10, 31.6, 100, 316, 1000 cd/m^2^ for 1 s, 1000 cd/m^2^ for 5 s, and 1000 cd/m^2^ for 10 s) are compared with MPCs induced by “Blue+Red” stimuli, where the same series of red light stimulations were presented at 9 s after 400 cd/m^2^, 200 ms blue light stimuli. The comparison of MPCs (mean±SD) is plotted in Fig. 1.

## 2 Experimental design, materials and methods

Study participants, testing protocols and data analysis method were described in the associated research article [1]. The chromatic pupillometry apparatus setup has also been previously described in detail [2], [3].
